# The effect of ketamine on affective modulation of the startle reflex and its resting-state brain correlates

**DOI:** 10.1038/s41598-023-40099-4

**Published:** 2023-08-16

**Authors:** Zümrüt Duygu Sen, Tara Chand, Lena Vera Danyeli, Vinod Jangir Kumar, Lejla Colic, Meng Li, Merve Yemisken, Nooshin Javaheripour, Alexander Refisch, Nils Opel, Tamar Macharadze, Moritz Kretzschmar, Esra Ozkan, Matthias Deliano, Martin Walter

**Affiliations:** 1 Department of Psychiatry and Psychotherapy, Jena University Hospital, Philosophenweg 3, 07743 Jena, Germany; 2 Clinical Affective Neuroimaging Laboratory (CANLAB), Magdeburg, Germany; 3 Department of Psychiatry and Psychotherapy, University Tübingen, Tübingen, Germany; 4 German Center for Mental Health (DZPG), Halle-Jena-Magdeburg Site, Jena, Germany; 5 Department of Clinical Psychology, Friedrich Schiller University Jena, Am Steiger 3-1, 07743 Jena, Germany; 6 Jindal Institute of Behavioural Sciences, O. P. Jindal Global University (Sonipat), Haryana, India; 7 Max Planck Institute for Biological Cybernetics, Tübingen, Germany; 8 Department of Anesthesiology and Intensive Care Medicine, Medical Faculty, Otto-Von-Guericke-Universität Magdeburg, Magdeburg, Germany; 9 Department Systems Physiology of Learning, Leibniz Institute for Neurobiology, Magdeburg, Germany; 10 Center for Behavioral Brain Sciences, Magdeburg, Germany; 11 Koç University Research Center for Translational Medicine, Istanbul, Turkey; 12 Leibniz Institute for Neurobiology, Magdeburg, Combinatorial NeuroImaging Core Facility, Brenneckestraße 6, 39118 Magdeburg, Germany; 13 Leibniz Institute for Neurobiology, Magdeburg, Germany

**Keywords:** Neuroscience, Emotion

## Abstract

Ketamine is a rapid-acting antidepressant that also influences neural reactivity to affective stimuli. However, the effect of ketamine on behavioral affective reactivity is yet to be elucidated. The affect-modulated startle reflex paradigm (AMSR) allows examining the valence-specific aspects of behavioral affective reactivity. We hypothesized that ketamine alters the modulation of the startle reflex during processing of unpleasant and pleasant stimuli and weakens the resting-state functional connectivity (rsFC) within the modulatory pathway, namely between the centromedial nucleus of the amygdala and nucleus reticularis pontis caudalis. In a randomized, double-blind, placebo-controlled, cross-over study, thirty-two healthy male participants underwent ultra-high field resting-state functional magnetic resonance imaging at 7 T before and 24 h after placebo and S-ketamine infusions. Participants completed the AMSR task at baseline and one day after each infusion. In contrast to our hypothesis, ketamine infusion did not impact startle potentiation during processing of unpleasant stimuli but resulted in diminished startle attenuation during processing of pleasant stimuli. This diminishment significantly correlated with end-of-infusion plasma levels of ketamine and norketamine. Furthermore, ketamine induced a decrease in rsFC within the modulatory startle reflex pathway. The results of this first study on the effect of ketamine on the AMSR suggest that ketamine might attenuate the motivational significance of pleasant stimuli in healthy participants one day after infusion.

## Introduction

Ketamine has revolutionized the treatment of major depressive disorder (MDD) by displaying an antidepressant effect within 24 h after a single application in subanesthetic doses^1^, while traditional antidepressants, serotonin reuptake inhibitors, show their effects within 3 to 4 weeks. Besides its general effect on depressive symptoms, ketamine also modulates neural affective processing^2–4^, which has been suggested to play a major role in pathophysiology and the treatment of MDD^5^. The affect-modulated startle reflex (AMSR) paradigm is a well-established translational paradigm to examine the behavioral aspect of affective reactivity. While MDD patients have a bias toward unpleasant stimuli in cognitive processes^6,7^, they show a blunted affective modulation of the startle reflex by both pleasant and unpleasant stimuli^8,9^. The intake of serotonin reuptake blockers might reinstate the affective modulation of the startle reflex in MDD patients^9^ and attenuate the modulation by unpleasant stimuli in healthy participants^10,11^. The effect of ketamine on the AMSR, however, remains to be elucidated.

The startle reflex is an evolutionary well-conserved brain stem reflex that is elicited by an unexpected stimulus. Lang and colleagues suggested that the AMSR is the result of an interaction between the motivational state (defensive or appetitive) induced by affective stimuli and the following activation of defensive circuits by the startling probes^12^. An unpleasant stimulus is suggested to serve as a cue that primes the defensive brain circuits that leads to a potentiation of the startle reflex. In contrast, a pleasant stimulus is suggested to activate the appetitive brain circuits, which results in a startle attenuation^13,14^. The AMSR is distinguished from other measures of affective reactivity, such as skin conductive response or pupil dilatation, which are rather arousal-specific^15^.

The primary acoustic startle reflex pathway begins with the nucleus cochlearis, which relays the auditory information to the nucleus reticularis pontis caudalis (PnC)^16^. The PnC then projects to motor neurons in the brainstem and spinal cord, which ultimately elicits the acoustic startle reflex. This primary startle pathway has been found to be modulated by respective cortical and subcortical regions exerting modulatory activity on the level of the PnC^16^. In line with this, the strength of the startle reflex may be modulated by affective, sensory, and attentional processes^14^. Animal studies showed that projections from the centromedial nucleus of the amygdala (CMA) to the PnC play a major role in the potentiation of the startle reflex^16,17^. Moreover, the potentiation of startle reflex was shown to be abolished in studies conducted with patients with amygdalar lesions^18–20^. However, for the startle reflex attenuation the underlying neuroanatomical pathways are largely unknown. It has been suggested that the nucleus accumbens and medial prefrontal cortex modulate the startle attenuation to appetitive or novel stimuli^21–23^ also relaying through the PnC^23^. Even though the amygdalar lesions did not affect pleasant modulation of the AMSR^22,24^, Anders and colleagues showed that bilateral amygdalar activity was associated with the startle reflex amplitude during affective picture viewing in healthy participants^25^, which was also supported by a recent study^26^. Taken together, studies indicated a central role of the CMA-PnC pathway in the affective modulation of the startle reflex.

The typical pattern across valence in the AMSR has been repeatedly reported to be abolished in MDD patients^8,9,27,28^, despite subjective valence ratings indicating a successful affect induction. Moreover, the decrease in unpleasant potentiation was associated with the severity of the depressive episode^29,30^. An exploratory study reported that the atypical affective modulation of the startle reflex did not recover after 2 weeks of SSRI treatment but recovered after 8 weeks in MDD patients^9^. There are various findings suggesting an effect of antidepressants on the AMSR also in healthy participants. One week of citalopram but not of reboxetine intake diminished the modulation of the AMSR by unpleasant stimuli^11^. Similarly, the unpleasant modulation of the AMSR was abolished 6 h after fluoxetine intake^10^. The effect of ketamine on the AMSR warrants further investigation but another non-competitive NMDA-R antagonist without antidepressant property, memantine, enhanced the unpleasant modulation compared to placebo in healthy participants^31^.

In the current randomized, double-blind, placebo-controlled, cross-over study, we investigated the effect of a single S-Ketamine infusion on the AMSR one day after infusion in a sample of healthy male participants. Since ketamine was shown to dampen the amygdalar processing of affective stimuli, particularly unpleasant stimuli, in the amygdala in MDD patients^4,32^, we hypothesized a decreased modulation by unpleasant pictures as well as pleasant pictures one day after S-ketamine infusion, at the time point of its peak antidepressant effect in MDD patients. Moreover, following the recent publication by Kuhn and colleagues (2020) showing the trial-by-trial association between the amplitude of the AMSR and blood-oxygen-level-dependent signal (BOLD) activation in the PnC and CMA for the first time in humans^26^, we examined the resting-state functional connectivity (rsFC) between the CMA and the PnC 24 h after infusion. In line with our hypothesis about the ketamine effect on the AMSR, we expected a ketamine-induced decrease in rsFC between the PnC and CMA.

## Methods

### Participants and study design

The data used in this study were collected as part of a bigger randomized, double-blind, placebo-controlled, cross-over study (SFB779/A06) that examined the immediate and delayed effects of a single S-Ketamine infusion. Thirty-five healthy male subjects (mean age ± standard deviation (SD) = 25.08 ± 4.18 years) were recruited via campus-wide and web-based advertisements. Medical examinations (physical examination, electrocardiogram, and blood and urine analyses) were carried out by study physicians. Psychiatric disorders were screened according to DSM-IV (Diagnostic and Statistical Manual of Mental Disorders, Fourth Edition; American Psychiatric Association^33^) by means of the Structured Clinical Interview for DSM-IV (SCID-I)^34^. None of the recruited subjects or their first-degree relatives had a current or lifetime history of a psychiatric disorder. Furthermore, none of the recruited subjects had a medical constraint that would contraindicate magnetic resonance imaging (MRI) or S-ketamine administration. This study was approved by the Institutional Review Board of the Otto-von-Guericke-University Magdeburg and was performed in accordance with the Declaration of Helsinki and local legal requirements. All the participants gave written informed consent prior to participation.

The study consisted of an initial measurement and two treatment arms with two consecutive measurement days. The first AMSR task was performed on the initial day (AMSR baseline session). The infusions were held on the second and fourth measurement days. Participants were randomly assigned to receive a placebo or ketamine infusion first. The time period between the two infusions was 3 weeks on average, with a minimum of 18 days to avoid carry-over effects. The infusion was administered as an initial bolus of S-Ketamine (0.11 mg/kg body weight) or saline over 8 min and a maintenance dose (0.22 mg/kg body weight S-Ketamine or 0.9% saline) over 40 min with a 2-min break between the bolus and maintenance infusions (Injectomat® MC Agilia; Fresenius Kabi GmbH, Bad Homburg, Germany). Vital signs were monitored during infusion (NONIN Pulse Oxymeter 8600-FO). The resting-state functional magnetic resonance imaging (rs-fMRI) at 7 T was measured directly before infusions (rsFC pre-placebo and pre-ketamine sessions) and during infusion on the second and fourth days as well as 24 h after infusions (rsFC post-placebo and post-ketamine sessions) on the third and fifth measurement days, which was followed by AMSR tasks outside the scanner (AMSR post-placebo and post-ketamine sessions) (sFig. 1A). In the current study, only pre- and post-infusion scans were analysed. Before and after MR scanning, additional behavioral tasks examining attention, interoception and interpretation bias as well as questionnaires were completed. Among others, a German version of the Positive and Negative Affect Schedule (PANAS; adapted from^35^) was completed for the assessment of changes in positive and negative affect. In short, in the current study, 3 AMSR task sessions (baseline, post-placebo, and post-ketamine), 4 rs-fMRI scanning sessions (pre-placebo, post-placebo, pre-ketamine, post-ketamine), and 4 self-reported questionnaires (pre-placebo, post-placebo, pre-ketamine, post-ketamine) were analyzed.

### Affect modulated startle reflex (AMSR) paradigm

The AMSR task was performed in an electrically shielded and soundproof chamber. Participants were given instructions on the paradigm and were told not to pay attention to the presented acoustic stimuli. Three different sets of color pictures were chosen from the International Affective Picture System (IAPS) based on their normative valence and arousal ratings (see Stable 1, 2, 3 for the complete list of pictures) for affect induction in each session. Each set of pictures included 14 pleasant (mean normative ratings with their standard deviations across sessions (as); valence_as = 7.23 ± 0.49 and arousal_as = 6.15 ± 0.57), 14 unpleasant (valence_as = 2.12 ± 0.54 and arousal_as = 6.45 ± 0.53) and 14 neutral pictures (valence_as = 5.00 ± 0.25 and arousal_as = 3.13 ± 0.92). The unpleasant pictures depict mutilation, animals such as snakes and spiders, or explosions. Pleasant pictures depict babies, food, and natural scenes but not erotic scenes. Neutral pictures depict objects. The three experimental picture sets did not differ from each other with respect to the published normative IAPS valence and arousal ratings (see Stable 4, Stable 5)^36^. The pictures were presented in a pseudorandomized order in a way that two pictures belonging to the same picture category (pleasant/neutral/unpleasant) did not follow each other. Preceding the presentation of experimental picture sets, three habituation pictures were shown in each session. The pictures were displayed for 6 s on a 29.4 cm color monitor placed at a distance of approximately 1 m in front of the participants. Each picture was followed by a 7-s interval assigned for the self-assessment manikin (SAM) assessment (sFigure 1B). The participants were asked to rate the valence and arousal of each picture on a digital form of the 9-point SAM rating scale^36^). To examine the ketamine effect on SAM ratings, the mean valence and arousal ratings were calculated for each picture category across trials per participant and per session.

The AMSR was induced by binaurally presented acoustic startle probes consisting of 50 ms white noise bursts with instantaneous rise times. Sounds were generated with a soundcard (Soundblaster Audigy Rx) at a sampling rate of 44,100 Hz and presented binaurally via a pair of free field stereo loudspeakers (50 W) positioned at a distance of about 1.2 m in front of the subjects. The sound intensity of the probe at the location of the subject’s head was between 88- and 95-dB (A) sound pressure level, as measured by a handheld sound level meter. The onset time of startle probes was varied randomly between 3500 and 4500 ms after the picture onset on display. To decrease the predictability of the startle probes, for one trial in each valence category, acoustic probes were presented during SAM ratings instead of during picture viewing and for another one trial in each category acoustic probes were omitted. In total, 12 pictures per valence category were accompanied by acoustic probes in each session. In the beginning of each session, the acoustic probes were delivered during the presentation of habituation sets of 3 pictures (one picture per category). The startle responses during the viewing of the habituation set were not included in the analysis. The pictures and acoustic probes were delivered by using the Psychophysics Toolbox extensions in version 3^37^.

### Physiological data recording and preprocessing

The startle electromyography (EMG) response was recorded bipolarly from two Ag/AgCl cup electrodes (1 cm diameter) placed over the lower orbital portion of the right orbicularis oculi muscle. Electrodes were filled with a conductive gel and attached to the skin by self-adhering washers and tape. Signals were recorded with a Brain Products ExG amplifier, digitized at 1 kHz sampling rate, and stored to disk. Offline preprocessing was performed using an in-house MATLAB script to filter and rectify the signal (see Supplement for details). Startle amplitude was defined as the difference between the peak startle and mean baseline amplitude (within 50 ms before probe onset). The raw startle amplitudes were then T-standardized (T_amp_, mean = 50, SD = 10) on the subject level for each session to reduce the inter- and intra-individual differences and increase the comparability across participants and across sessions^37^. To examine the ketamine effect on AMSR, the mean T_amp_ was calculated for each picture category across trials per participant and per session. Out of the 35 subjects, three subjects did not perform AMSR paradigm, resulting in 32 participants (age: 25.25 $\pm$ 4.30) that were included in the current analysis.

### Magnetic resonance imaging acquisition

All data (structural and resting state functional MRI) were acquired on a Siemens 7 T scanner (Siemens Healthineers, Erlangen, Germany) in Magdeburg. Following the automated shimming, T1-weighted structural images were acquired using a magnetization–prepared rapid gradient–echo (MPRAGE) sequence with the following parameters: repetition time (TR) = 1700 ms, echo time (TE) = 2.54 ms, inversion time (TI) = 1050 ms, flip angle = 5°, 176 slices, field of view (FoV) = 256 mm, grappa acceleration factor PE = 2, bandwidth = 160 Hz/pixel, isotropic voxel size = 1 mm^3^. After the field maps sequence, the rsfMRI data were acquired using an echo-planar imaging (EPI) sequence with the following settings: TR = 1500 ms, TE = 25 ms, flip angle = 70°, 60 slices, FoV = 212 mm, isotropic voxel size = 2 mm^3^, multi-band acceleration factor = 3, grappa acceleration factor PE = 2. All subjects were instructed to keep their eyes closed, not to think about anything specific, and not to fall asleep during the resting-state measurements.

### Functional connectivity between PnC and CMA

After preprocessing the data (see Supplement for the details), we manually drew a right PnC mask around the peak coordinate (4 − 34 − 37). The PnC nuclei definition relies on the cluster map, i.e., peak coordinate (4 − 34 − 37) presented in the seminar work by Kuhn et al.^25^. Instead of drawing a sphere around the peak coordinate, we took into consideration the cylindrical shape of the PnC nucleus (Duvernoy's Atlas of the Human Brain Stem and Cerebellum' 2009). By visually matching the atlas depiction of nuclei boundaries and shape and following the nuclei boundaries outlined in the atlas 'Duvernoy's Atlas of the Human Brain Stem and Cerebellum' (2009), we created the right PnC mask. Furthermore, a corresponding left PnC mask was generated using the FSL flip command. (SFig. 2). Afterwards, the left and right CMA masks were used from the SPM anatomical toolbox (JuBrain Anatomy Toolbox v3.0). The quality check was performed visually by overlaying ROIs on the mean EPI functional images of each subject per each session. The mean time series were extracted and rsFC was calculated by Pearson correlation. The correlation coefficients were Fisher Z-transformed. Of note, five participants who exceeded a mean FD of 0.25 mm in one of the sessions were excluded from the analysis. In total, 30 subjects were included in the rsfMRI region of interest analysis.

### Blood levels of ketamine and its metabolites

Venous blood samples were taken from the non-infusion arm at 8, 13, and 48 min after the beginning of the infusion. Blood samples were kept on ice, processed, and freezed within 2 h after the sampling. The samples were analyzed with liquid chromatography hyphenated targeted mass spectrometry for the simultaneous analysis of ketamine (KET), and norketamine (NK (for details, see^38^. KET and NK 48 min were available for all participants and were therefore used for subsequent correlation analyses.

### Statistical analysis

The effect of ketamine infusion on the scores of the Positive and Negative Affect Schedule (PANAS) was examined by a linear mixed model analysis using *Session* (post-placebo and post-ketamine) and session-specific baseline scores as fixed terms in a linear mixed model. A subject-specific random intercept was modeled, and the baseline scores were allowed to vary between subjects.

To examine the ketamine effect on SAM ratings and AMSR, the mean valence and arousal ratings, as well as T_amp_, were calculated for each picture category across trials per participant and per session. For each variable, a linear mixed model with the factors *Session* (baseline, post-placebo, and post-ketamine) and *Picture Category* (pleasant, neutral, and unpleasant) as well as their interaction as fixed effects and *Picture Category* by *Participant* as random intercept was fitted. Significant interaction effects were followed by paired sample t-tests and p-values were adjusted by Bonferroni correction for within sessions comparisons. Additional linear mixed models were fitted to examine the effect of the drug order and age. To examine the valence-specific effect of treatment on affective modulation, *unpleasant modulation* (T_amp_ in unpleasant category minus T_amp_ in neutral category) and *pleasant modulation* (T_amp_ in pleasant category minus T_amp_, in neutral category) values were calculated for each session. Linear mixed models were fitted to test the effect of *Session* (baseline, post-placebo, and post-ketamine) including *Participant* as random intercept. The Bonferroni correction was used to adjust p-values for posthoc comparisons. The difference between *modulation* scores in post-ketamine and baseline sessions was calculated and the association between the ketamine-induced change in *modulation* and the plasma levels of ketamine and norketamine was tested by Pearson correlation.

To examine the ketamine effect on rsFC between CMA and PnC, a linear mixed model was applied. *Session* (post-placebo and post-ketamine) and session-specific pre-infusion rsFC were used as fixed terms. A subject-specific random intercept was modeled and the pre-infusion rsFC was allowed to vary between subjects. For visualization purposes, pre-infusion and post-infusion rsFCs were compared by paired sample t-tests for placebo and ketamine arms separately. Additional Pearson correlations were run to examine the association between the ketamine-induced change in rsFC and plasma levels as well as ketamine-induced change in AMSR.

All statistical analyses were performed, and figures were generated using R (version 3.6.3) and the package ggplot2 (version 3.3.3). Linear mixed models were implemented by using the “nlme” library in R version 3.6.3. For all models, an unstructured variance–covariance matrix was used. Normality and homogeneity of the variance were checked via examination of residual plots. Models’ fits were compared by using the anova function from stats packages^39^. Estimates were obtained by restricted maximum likelihood estimation. The marginal means of model estimates and their contrasts were calculated with the emmeans package^40^. The alpha value was set to 0.05.

## Results

### Self-reported unpleasant and pleasant affect scores

The effect of ketamine administration on positive and negative affect was examined by running separate linear mixed models on PANAS scores 24 h after infusion (STable 6, STable 7). There was no significant effect of *Session* (post-placebo, post-ketamine) on positive (p = 0.55) and negative affect scores (p = 0.21).

### Subjective valence and arousal ratings of affective pictures

There was a significant effect of the *Picture Category* (pleasant, neutral, and unpleasant) on subjective valence ratings (p’s > 0.001, see STable 10). The effect of *Session* (baseline, post-placebo, and post-ketamine) and *Session-by-Picture Category* interaction were not statistically significant (all p’s < 0.05, see STable 10). In each session, pleasant pictures were rated higher in valence than neutral and unpleasant pictures, while unpleasant pictures were rated lower than neutral pictures (all Bonferroni adjusted p (p_adj_)’s < 0.001). There also was a significant effect of the *Picture Category* on arousal ratings (p’s < 0.001, see STable 11) but no effect of Session or *Session-by-Picture Category* interaction (all p’s < 0.05, see STable 11). Post hoc comparisons showed that unpleasant pictures were rated higher in arousal than pleasant and neutral pictures, while pleasant pictures were rated higher than neutral pictures in all sessions (all p_adj_’s < 0.001).

### Affective modulation of the startle reflex amplitudes

**Figure 1:**
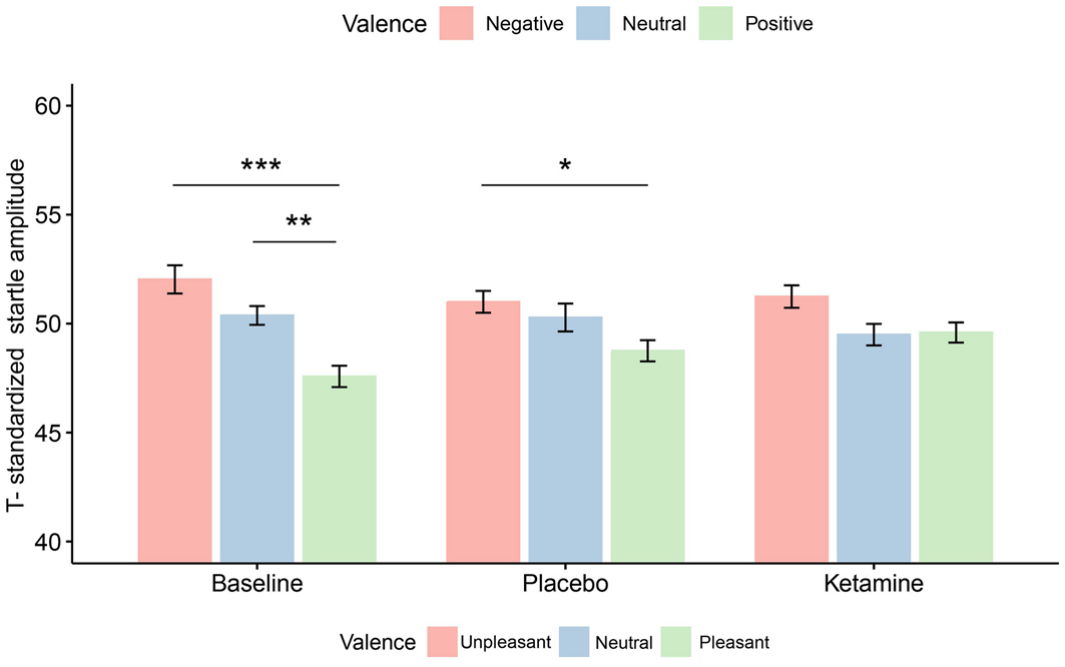
T-standardized startle reflex amplitudes for each picture category and session. Pleasant pictures elicited a significantly less startle reflex amplitude relative to unpleasant pictures in the baseline (T = 6.00, padj < 0.001) and post-placebo sessions (T = 3.09, padj = 0.02) but not in post-ketamine session (T = 2.21, padj = 0.21). Compared to neutral pictures, pleasant pictures elicited a less startle reflex amplitude only in the baseline session (T = 3.81, padj = 0.002). Error bars represent ± 1 standard error of the mean.; *p < 0.05; **p < 0.01; ***p < 0.001.

There was a significant effect of *Picture Category* (pleasant, neutral, and unpleasant) and *Session-by-Picture Category* interaction (Table 1) on the T_amp_ one day after the infusion. Overall, negative pictures evoked a more pronounced startle reflex amplitude compared to neutral pictures (p = 0.03) and pleasant pictures (p < 0.001). As a follow-up, the mean T_amp_ for each session was compared across picture categories (Fig. 1). Pleasant pictures evoked a significantly less pronounced startle reflex amplitude relative to unpleasant pictures in the baseline (Estimate (*b)* = 4.49, standard error (SE) = 0.75, p_adj_ < 0.001) and post-placebo sessions (*b* = 2.34, SE = 0.76, p_adj_ = 0.02) but not in post-ketamine session (*b* = 1.64, SE = 0.74, p_adj_ = 0.21). The T_amp_ did not differ between unpleasant and neutral picture categories in none of the sessions and the pleasant pictures evoked a less pronounced startle reflex amplitude relative to neutral pictures significantly only in the baseline session (*b* = 2.79, SE = 0.73, p_adj_ = 0.002).

**Table 1:** The linear mixed model fixed effect estimates of Picture Category and Session on T-normalized startle amplitudes.

	B	CI (95%)	T	p	df
(Intercept)	52.05	51.00–53.10	97.80	**< 0.001**	237.00
Session [ketamine]	− 0.82	− 2.19–0.56	− 1.17	0.24	237.00
Session [placebo]	− 0.97	− 2.36–0.42	− 1.38	0.17	237.00
*Picture category* [neutral]	− 1.69	− 3.23 to − 0.15	− 2.16	**0.03**	237.00
*Picture category* [pleasant]	− 4.48	− 5.95 to − 3.01	− 6.01	** < 0.001**	237.00
Session [ketamine] * *Picture category* [neutral]	− 0.05	− 1.99–1.89	− 0.05	0.96	237.00
Session [placebo] * *Picture category* [neutral]	0.82	− 1.15–2.80	0.82	0.41	237.00
Session [ketamine] * Picture category [pleasant]	2.84	0.90–4.78	2.89	** < 0.01**	237.00
Session [placebo] * Picture-category [pleasant]	2.14	0.18–4.11	2.15	**0.03**	237.00
Observations	277				
Marginal R^2^/conditional R^2^	0.166/0.252				

Significant values are in bold.

*B* Estimates, *CI* confidence interval; Reference category for Session was Baseline and for Picture category was Unpleasant.

**Figure 2:**
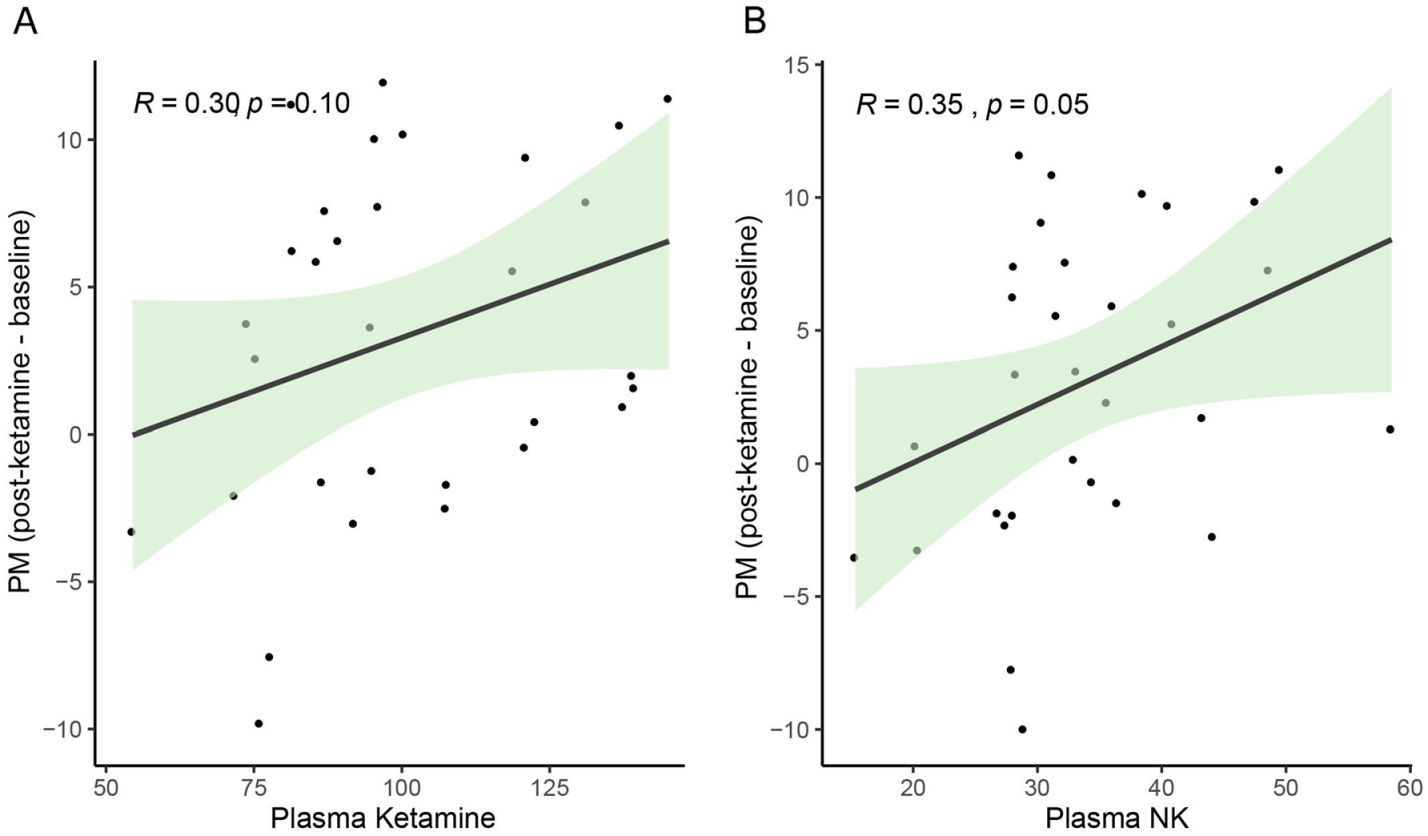
The association between the ketamine-induced change in pleasant modulation (pleasant-neutral) and end-of-infusion plasma levels of ketamine (**A**) and its metabolite norketamine (**B**). The shaded area around the line represents the 95% confidence interval.

To examine the effect of treatment on valence-specific affective modulation, *unpleasant modulation* (difference in T_amp_ between unpleasant and neutral categories), and *pleasant modulation* (difference in T_amp_ between neutral and pleasant categories) values were calculated for each session. There was no significant effect of *Session* (baseline, post-placebo, and post-ketamine) on *unpleasant modulation* (STable 12), while a significant effect of *Session* was found for *pleasant modulation* (STable 13)*.* Post hoc comparisons showed that there was a decrease in *pleasant modulation* in post-ketamine session compared to baseline (*b* = 2.91, SE = 1.02, p_adj_ = 0.02), but not in post-placebo session (*b* = 1.09, SE = 1.33, p_adj_ = 1.000). The decrease in *pleasant modulation* post-ketamine showed a rough positive trend and significant correlation with the end-of-infusion blood levels of ketamine (r = 0.30, p = 0.10) and norketamine (r = 0.35, p = 0.05), respectively (Fig. 2).

### The rsFC between the CMA and PnC

**Figure 3:**
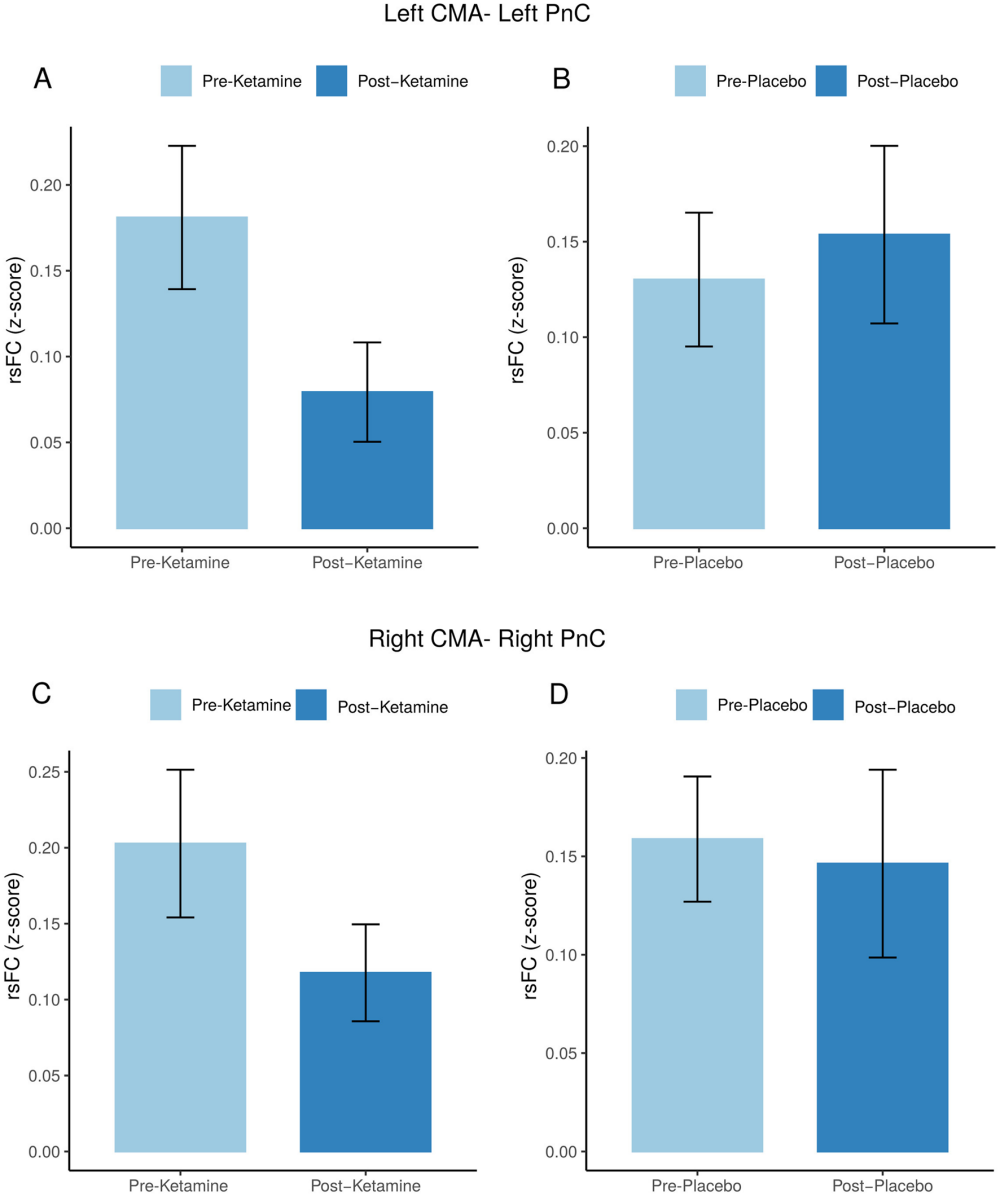
The effect of treatment on rsFC in modulatory of startle pathways. RsFC in left CMA and PnC pre- and post-ketamine infusion (**A**) and pre- and post-placebo infusion (**B**). RsFC in right CMA and PnC pre- and post-ketamine infusion (**C**) and pre- and post-placebo infusion (**D**).

There was a significant effect of *Session* (post-placebo and post-ketamine) on rsFC between left CMA and left PnC 24 h after infusion (p = 0.04, Table 2) but not on the right side (p = 0.41). Follow-up analyses showed that rsFC between CMA and PnC decreased after ketamine infusion relative to baseline on the left side (T (29) = 1.92, p = 0.06). However, a similar trend in rsFC change relative to pre-infusion values was seen on both sides (Fig. 3). The ketamine-induced change in rsFC between left CMA and left PnC did not show a significant correlation with plasma levels of ketamine (r = − 0.26; p = 0.16) or norketamine (r = − 0.30; p = 0.11).

**Table 2:** The linear mixed model fixed effect estimates of Session on rsFC between CMA and PnC.

	B	CI (95%)	T	p	df
CMA-PnC (left)					
Intercept	0.06	− 0.03–0.16	1.33	0.19	29
Session [placebo]	0.09	0.00–0.17	2.10	**0.04**	28
Baseline	0.06	− 0.19–0.32	0.52	0.60	28
Observations	60				
Marginal R^2^/conditional R^2^	0.04/0.74				
CMA-PnC (right)					
Intercept	0.09	− 0.00–0.18	2.02	**0.05**	29
Session [placebo]	0.03	− 0.05–0.12	0.80	0.42	28
Baseline	0.12	− 0.12–0.37	1.05	0.30	28
Observations	60				
Marginal R^2^/conditional R^2^	0.02/0.73				

Significant values are in bold.

*CMA* centromedial nucleus of the amygdala, *PnC* nucleus reticularis pontis caudalis, *B* Estimates, *CI* confidence interval.

Additional correlations were run to examine the association between the ketamine-induced alteration in rsFC and ASMR. The ketamine-induced change in rsFC relative to baseline was not correlated with ketamine-induced changes in neither unpleasant (p = 0.85) nor pleasant modulation (p = 0.25).

## Discussion

In this placebo-controlled cross-over study in healthy male participants, we showed that a single S-ketamine administration resulted in an atypical pattern of the AMSR one day after infusion, mainly driven by the decreased modulation of the startle reflex by pleasant pictures compared to baseline. While an attenuated startle reflex amplitude was seen during the presentation of pleasant pictures relative to neutral pictures at baseline, this pattern was abolished 24 h after ketamine infusion. The decrease in pleasant modulation showed a positive correlation with the end-of-infusion plasma levels of ketamine's metabolite norketamine, supporting that this effect was induced by the ketamine administration. Based on the recent literature highlighting the role of the PnC and CMA in the AMSR in humans^26^, we examined the effect of ketamine on the rsFC between these two regions 24 h after ketamine infusion. Our results showed that the rsFC between the left PnC and left CMA decreased significantly after ketamine. Interestingly, these effects of ketamine on the AMSR and its neural pathways were not accompanied by changes in the reported levels of general positive and negative affect or in the valence and arousal ratings of affective pictures, which is in line with the literature^9–11^. Our results, therefore, suggest that ketamine may blunt the motivational significance of pleasant stimuli without an effect on the conscious evaluation of the affect induced by the stimulus and without changing the general affect. Since the effects of antidepressants on affective processing might precede and underlie their effect on depressive mood^41^ the present investigation of healthy individuals might broaden our understanding of their neuropsychological mechanism of action. Current evidence suggests that antidepressants, including ketamine, may dampen the processing of unpleasant stimuli in healthy participants^11,42,43^. In line with this, some studies showed that intake of citalopram and fluoxetine decreased the unpleasant modulation of the AMSR after a single use^10,11^. Therefore, we hypothesized that ketamine decreases the unpleasant modulation of the AMSR. On the contrary, we did not see a significant difference in the startle reflex modulation by unpleasant pictures after ketamine infusion. Our result is in line with the view that the effect of antidepressants on AMSR may depend on its pharmacodynamic properties rather than on direct decrease in depressive mood. For example, while citalopram, agomelatine, and fluoxetine decreased the unpleasant modulation^10,11,44^, reboxetine, duloxetine, and mirtazapine did not^11,42,45^. Moreover, most studies investigate different time point(s) after agent administration and use cross-sectional design indicating that more research is needed to elucidate dynamics of AMRS induced by antidepressant agents.

In the current study, the pleasant modulation was attenuated one day after ketamine administration, while there was a positive correlation with the plasma levels of norketamine. Another antidepressant agent that abolishes the pleasant modulation is agomelatine, a melatonergic receptor agonist and 5HT2C receptor antagonist^44^. In contrast, the effect of SSRIs on pleasant modulation has not been reported. It has been shown that ketamine suppresses the processing of pleasant stimuli during infusion in healthy participants^46–48^, but its effect at the time point of the peak antidepressant response (24 h after administration) remains less clear. Studies performed on MDD patients suggest that ketamine facilitates the neural processing of pleasant stimuli 1–2 days after infusion^3,4,32^. However, findings regarding the AMSR are not directly comparable to many other affective processing tasks that are more associated with other domains of affective reactivity, such as cognition or reward sensitivity.

In line with our hypothesis, we found that rsFC in the modulatory pathways of the startle, namely between left PnC and left CMA, decreased 24 h after ketamine administration. This effect was also evident bilaterally, though not statistically significant. Moreover, the association between ketamine-induced decrease in rsFC and plasma norketamine levels showed a rough negative trend. The PnC receives information from cochlear root neurons and serves as a hub that connects primary and modulatory startle reflex pathways^16^, while the CMA plays a central role in estimating the motivational significance of both unpleasant and pleasant stimuli^49^. We also tested if the ketamine-induced decrease in rsFC in modulatory circuits correlates with the decrease in pleasant modulation of the AMSR. However, there was no significant correlation between these two effects of ketamine. This lack of association might be due to the fact that our study only acquired resting-state measures. Task-fMRI studies focusing on the effect of ketamine on pathways of the AMSR are needed to support the current findings and further examine the ketamine effect. Alternatively, the positive modulation can be more related to the effect of other brain regions such as nuclues accumbens on PnC^16,23^. However, the role of nucleus accumbens on positive modulation has not been shown in humans yet. Therefore, we limited our analysis to CeA and PnC, of which role was shown in humans recently^26^.

Although we could show a decrease in the rsFC within the modulatory pathway after ketamine application, there was no ketamine effect on unpleasant modulation in the AMSR task. As we discussed above, it is plausible that ketamine might show no effect on the motivational processing of unpleasant stimuli, which might be explained by its pharmacodynamic properties. However, our results bear an interesting parallel to other studies in the literature that reported changes in neuronal processing of emotional stimuli after ketamine without a ketamine effect on task performance^2,47,50,51^. In a study with healthy volunteers decreased neural reactivity to the negative stimuli was detected during ketamine infusion, while the performance in an affective memory test was not affected^52^. Another study using attentional dot probe task with emotional faces showed no effect of ketamine on task performance 2 days after infusion in MDD patients, while a greater neural activation to angry vs happy faces was observed, a pattern similar to post-placebo measurements of healthy controls^53^). Moreover, it is likely that the effect of antidepressants on behavior might not be evident in healthy individuals with typical task performance at baseline, while subtle differences induced by treatment can be relatively easier to detect with neuroimaging techniques. While it is beneficial to examine the antidepressant drug action in healthy participants since it provides an opportunity to focus on their effect on affective reactivity without the confounding effect of depressed mood on affective processes^41^, future studies that also include MDD patients would broaden the scope of understanding of the current findings also in a clinical context. Of note, during infusion, ketamine might increase the neuromuscular reactivity^54–56^. However, startle reflex amplitude was shown to be decreased during ketamine infusion in healthy volunteers^57,58^; but no effect was shown by Duncan et al.^59^ while the habituation was not affected^57,58^. To our knowledge this is the first report of the effects 24 h after ketamine infusion. In the current study, ketamine did not effect overall startle reactivity and habituation profiles (See Supplementary Results).

Several limitations need to be mentioned. Firstly, startle responses were induced by speakers instead of headphones, and the amplitude of the stimulus ranged between 85 to 95 dB(A). Based on the high rate of missing startle responses, it is possible that we might not have reliably induced startle responses. However, the probability of a startle response did not differ between picture categories and sessions (see STable 12), excluding a systematic bias affecting only one picture category or one treatment arm. Furthermore, we limited our sample to healthy male participants. Future studies with larger samples, including female participants and MDD patients, are needed to have a more detailed understanding of the effect of ketamine on affective reactivity. An important point to mention is that the linear mixed model with raw startle reflex amplitudes did not converge. This is likely due to the large between and within participant variation in startle reflex amplitudes, which is in line with the literature^37^. Therefore, following the literature the raw startle amplitudes were T-standardized in the current study. The reasons behind the large variation in startle reflex amplitudes are yet to be determined. Micro-variations in the experimental procedures such as the placement of the electrodes might be one of the reasons. The failed replication of the main results with raw startle reflex amplitudes might reduce the generalizability of the results. Of note, during infusion, ketamine might increase the neuromuscular reactivity^54–56^. However, startle reflex amplitude was shown to be decreased during ketamine infusion in healthy volunteers^57,58^; but no effect was shown by Duncan et al.^59^ while the habituation was not affected^57,58^. To our knowledge this is the first report of the effects 24 h after ketamine infusion. In the current study, ketamine did not effect overall startle reactivity and habituation profiles (See Supplementary Results). Another point that should be taken into consideration is that the MRS scans were held at rest and the effect of ketamine on reactivity of the amygdala and PnC could not be assessed. Yet, the fingerprints of the interaction between brain regions during a task might also be observed at the resting state^60,61^. However, task-fMRI studies are needed for the optimal assessment of ketamine's effect on the modulatory pathway of the startle reflex.

Of note, this study relies on the standard confounding regression of physiological noise in the fMRI data. However, brainstem nuclei are more prone to be affected by motion and physiological noise (respiration and pulse-oxygen/heart rate). Recording these brainstem-specific physiological signals would help in future studies to delineate a more reliable view of the presented results. Lastly, despite the study being double-blind, many participants were able to distinguish between the ketamine and placebo infusions due to the evident subjective effects produced by ketamine. This situation highlights the challenge of maintaining complete blinding when studying substances with distinct psychoactive properties.

In conclusion, a single S-ketamine infusion decreased the rsFC within the modulatory pathway of the AMSR and pleasant modulation of the AMSR without an effect on unpleasant modulation. These results indicate an effect of ketamine on motivational and affective processing in healthy participants. This may help to understand the mechanism of drug action not only in MDD but also in many other mental disorders, such as addiction and anxiety disorders, which show an atypical AMSR pattern^62,63^ as well as a treatment response to ketamine^64,65^.

### Supplementary Information

Supplementary Information.

## Data Availability

The datasets generated during and/or analysed during the current study are available from the corresponding author on reasonable request.
