# Author Correction: The effect of ketamine on affective modulation of the startle reflex and its resting-state brain correlates

**DOI:** 10.1038/s41598-023-42343-3

**Published:** 2023-09-22

**Authors:** Zümrüt Duygu Sen, Tara Chand, Lena Vera Danyeli, Vinod Jangir Kumar, Lejla Colic, Meng Li, Merve Yemisken, Nooshin Javaheripour, Alexander Refisch, Nils Opel, Tamar Macharadze, Moritz Kretzschmar, Esra Ozkan, Matthias Deliano, Martin Walter

**Affiliations:** 1 Department of Psychiatry and Psychotherapy, Jena University Hospital, Philosophenweg 3, 07743 Jena, Germany; 2 Clinical Affective Neuroimaging Laboratory (CANLAB), Magdeburg, Germany; 3 Department of Psychiatry and Psychotherapy, University Tübingen, Tübingen, Germany; 4 German Center for Mental Health (DZPG), Halle-Jena-Magdeburg Site, Jena, Germany; 5 Leibniz Institute for Neurobiology, Magdeburg, Germany; 6 Max Planck Institute for Biological Cybernetics, Tübingen, Germany; 7 Department of Anesthesiology and Intensive Care Medicine, Medical Faculty, Otto-Von-Guericke-Universität Magdeburg, Magdeburg, Germany; 8 Department Systems Physiology of Learning, Leibniz Institute for Neurobiology, Magdeburg, Germany; 9 Koç University Research Center for Translational Medicine, Istanbul, Turkey; 10 Center for Behavioral Brain Sciences, Magdeburg, Germany; 11 Leibniz Institute for Neurobiology, Magdeburg, Combinatorial NeuroImaging Core Facility, Brenneckestraße 6, 39118 Magdeburg, Germany; 12 Department of Anesthesiology and Intensive Care Medicine, OVGU, Magdeburg, Germany; 13 Department of Clinical Psychology, Friedrich Schiller University Jena, Am Steiger 3-1, 07743 Jena, Germany; 14 Jindal Institute of Behavioural Sciences, O. P. Jindal Global University (Sonipat), Haryana, India

Correction to: *Scientific Reports* 10.1038/s41598-023-40099-4, published online 16 August 2023

The original version of this Article omitted affiliations for Tara Chand. The correct affiliations are listed below.

Department of Psychiatry and Psychotherapy, Jena University Hospital, Philosophenweg 3, 07743, Jena, Germany

Clinical Affective Neuroimaging Laboratory (CANLAB), Magdeburg, Germany

Department of Clinical Psychology, Friedrich Schiller University Jena, Am Steiger 3-1, 07743, Jena, Germany

Jindal Institute of Behavioural Sciences, O. P. Jindal Global University (Sonipat), Haryana, India

The original Article has been corrected.

