# Supplementary material for: The effect of ketamine on affective modulation of the startle reflex and its resting-state brain correlates
Source: Sci Rep. 2023 Aug 16;13:13323. doi: 10.1038/s41598-023-40099-4 (PMC10432502; doi:10.1038/s41598-023-40099-4)
Supplement: Supplementary file 1 — Supplementary Information. [file 41598_2023_40099_MOESM1_ESM.docx]

**Supplementary material**

**Supplementary Methods**

**
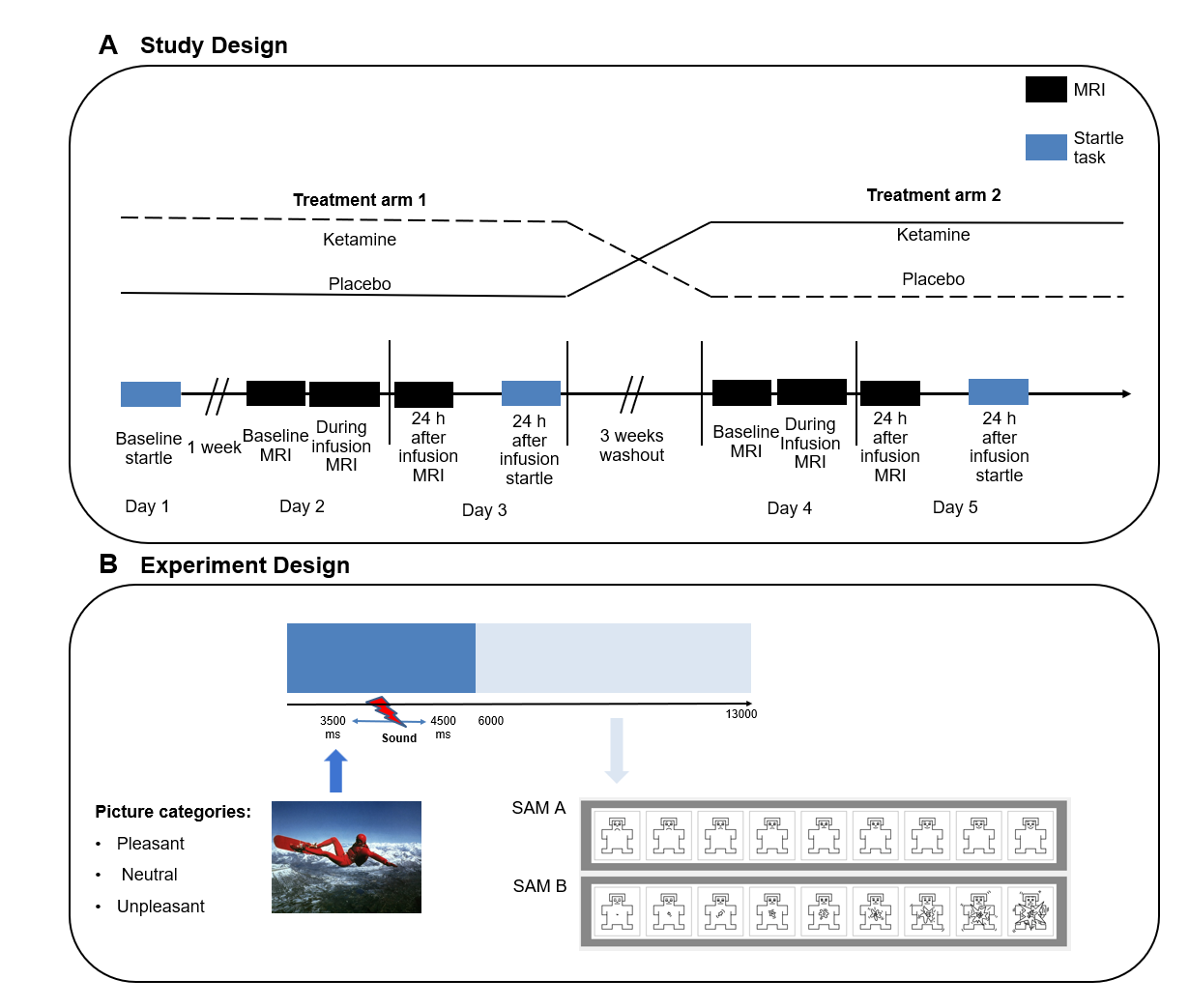
**

**SFigure 1** **A**) Study Design. **B**) Affect-modulated startle reflex paradigm

**STable 1**

*The set of IAPS pictures used in the session-1 and their normative valence and arousal ratings.*

| Pleasant | | | Neutral | | | Unpleasant | | |
| --- | --- | --- | --- | --- | --- | --- | --- | --- |
| ID | V | A | ID | V | A | ID | V | A |
| 8170 | 7,63 | 6.12 | 7020 | 4.97 | 2.17 | 9630 | 2.96 | 6.06 |
| 4597 | 6.95 | 5.91 | 7950 | 4.94 | 2.28 | 9300 | 2.26 | 6 |
| 2045 | 7.87 | 5.47 | 7006 | 4.88 | 0.99 | 3170 | 1.46 | 7.21 |
| 8186 | 7.01 | 6.84 | 7217 | 4.82 | 2.43 | 9410 | 1.51 | 7.07 |
| 7501 | 6.85 | 5.63 | 7043 | 5.17 | 3.68 | 3500 | 2.21 | 6.99 |
| 8501 | 7.91 | 6.44 | 7287 | 4.77 | 3.57 | 6300 | 2.59 | 6.61 |
| 2209 | 7.64 | 5.59 | 7175 | 4.87 | 1.72 | 3550 | 2.54 | 5.92 |
| 4645 | 6.73 | 6.61 | 7002 | 4.97 | 3.16 | 9413 | 1.76 | 6.81 |
| 8490 | 7.2 | 6.68 | 2516 | 4.9 | 3.5 | 3110 | 1.79 | 6.7 |
| 7270 | 7.53 | 5.76 | 6150 | 5.08 | 3.22 | 9908 | 2.34 | 6.63 |
| 4598 | 6.33 | 5.53 | 2441 | 4.64 | 3.62 | 9592 | 3.34 | 5.23 |
| 5626 | 6.71 | 6.09 | 7188 | 5.5 | 4.28 | 9635 | 1.9 | 6.54 |
| 7230 | 7.38 | 5.52 | 5395 | 5.34 | 4.23 | 3168 | 1.56 | 6 |
| 8030 | 7.33 | 7.35 | 7484 | 4.99 | 4.24 | 3102 | 1.4 | 6.58 |

V: Valence, A: Arousal

**STable 2**

*The set of IAPS pictures used in the session-2 and their normative valence and arousal ratings.*

| Pleasant | | | Neutral | | | Unpleasant | | |
| --- | --- | --- | --- | --- | --- | --- | --- | --- |
| ID | V | A | ID | V | A | ID | V | A |
| 8492 | 7.21 | 7.31 | 7547 | 5.21 | 3.18 | 2981 | 2.76 | 5.97 |
| 7502 | 7.75 | 5.91 | 7513 | 5.45 | 3.47 | 9075 | 1.66 | 6.04 |
| 8193 | 6.73 | 6.04 | 2309 | 4.89 | 1.71 | 3120 | 1.56 | 6.84 |
| 4640 | 7.18 | 5.52 | 7710 | 5.42 | 3.44 | 9433 | 1.84 | 5.89 |
| 8179 | 6.48 | 6.99 | 7500 | 5.33 | 3.26 | 2691 | 3.04 | 5.85 |
| 8502 | 7.51 | 5.78 | 7187 | 5.07 | 1.02 | 3005.1 | 1.63 | 6.2 |
| 5833 | 8.22 | 5.71 | 7110 | 4.55 | 2.27 | 6230 | 2.37 | 7.35 |
| 4643 | 6.84 | 6.01 | 7058 | 5.29 | 3.98 | 9325 | 1.89 | 6.01 |
| 8163 | 7.14 | 6.53 | 7057 | 5.35 | 3.39 | 3080 | 1.48 | 7.22 |
| 7650 | 6.62 | 6.15 | 7044 | 4.69 | 3.94 | 9250 | 2.57 | 6.6 |
| 2347 | 7.83 | 5.56 | 7031 | 4.52 | 2.03 | 6570 | 2.19 | 6.24 |
| 4599 | 7.12 | 5.69 | 7590 | 4.75 | 3.8 | 3010 | 1.79 | 7.26 |
| 5621 | 7.57 | 6.99 | 2446 | 4.7 | 3.79 | 2683 | 2.62 | 6.21 |
| 1650 | 6.65 | 6.23 | 2279 | 4.71 | 3.74 | 3063 | 1.49 | 6.35 |

V: Valence, A: Arousal

**STable 3**

*The set of IAPS pictures used in the session-3 and their normative valence and arousal ratings.*

| Pleasant | | | Neutral | | | Unpleasant | | |
| --- | --- | --- | --- | --- | --- | --- | --- | --- |
| ID | V | A | ID | V | A | ID | V | A |
| 8370 | 7.77 | 6.73 | 2840 | 4.91 | 2.43 | 9902 | 2.33 | 6 |
| 2216 | 7.57 | 5.83 | 2377 | 5.19 | 3.5 | 6213 | 2.91 | 5.86 |
| 8251 | 6.16 | 6.05 | 8312 | 5.37 | 3.32 | 9940 | 1.62 | 7.15 |
| 4626 | 7.6 | 5.78 | 7487 | 4.92 | 4.08 | 3261 | 1.82 | 5.75 |
| 5629 | 7.03 | 6.55 | 7000 | 5 | 2.42 | 9321 | 2.81 | 6.24 |
| 5260 | 7.34 | 5.71 | 7004 | 5.04 | 2 | 3266 | 1.56 | 6.79 |
| 8178 | 6.5 | 6.82 | 7016 | 4.76 | 3.4 | 3000 | 1.59 | 7.34 |
| 5700 | 7.61 | 5.68 | 1645 | 4.99 | 1.64 | 6315 | 2.31 | 6.38 |
| 8499 | 7.63 | 6.07 | 7550 | 5.27 | 3.95 | 3060 | 1.79 | 7.12 |
| 8500 | 6.96 | 5.6 | 7018 | 4.81 | 3.91 | 9921 | 2.04 | 6.52 |
| 8080 | 7.73 | 6.65 | 7242 | 5.28 | 3.83 | 9909 | 2.78 | 5.98 |
| 8185 | 7.57 | 7.27 | 7010 | 4.94 | 1.07 | 9405 | 1.83 | 6.08 |
| 5470 | 7.35 | 6.02 | 5535 | 4.81 | 4.11 | 3195 | 2.06 | 6.36 |
| 7570 | 6.97 | 5.54 | 7036 | 4.88 | 3.32 | 3213 | 2.96 | 6.82 |

V: Valence, A: Arousal

**STable 4**

*Comparison of the mean normative valence ratings between IAPS picture sets across picture sets*

|  | Set-1  Mean$\pm$SD | Set-2  Mean$\pm$SD | Set-3  Mean$\pm$SD | F | p |
| --- | --- | --- | --- | --- | --- |
| Pleasant | 7.22$\pm$0.47 | 7.20$\pm$0.51 | 7.27$\pm$ 0.48 | 0.07 | 0.93 |
| Neutral | 4.99$\pm$ 0.22 | 4.50$\pm$0.34 | 5.01$\pm$0.19 | 0.03 | 0.97 |
| Unpleasant | 2.11$\pm$0.59 | 2.06$\pm$0.52 | 2.17$\pm$0.51 | 0.14 | 0.87 |

SD: standard deviation

**STable 5**

*Comparison of normative arousal ratings between selected IAPS picture sets*

|  | Set-1  Mean$\pm$SD | Set-2  Mean$\pm$SD | Set-3  Mean$\pm$SD | F | p |
| --- | --- | --- | --- | --- | --- |
| Pleasant | 6.11$\pm$0.59 | 6.17$\pm$0.57 | 6.16$\pm$0.54 | 0.05 | 0.95 |
| Neutral | 3.08$\pm$1.01 | 3.07$\pm$0.93 | 3.07$\pm$0.98 | 0.00 | 1.00 |
| Unpleasant | 6.45$\pm$0.54 | 6.43$\pm$0.53 | 6.46$\pm$0.51 | 0.01 | 0.99 |

SD: standard deviation

**Physiological data preprocessing**

Offline preprocessing was performed using an in-house MATLAB script to filter and rectify the signal. A band-pass (24-490 Hz) and a notch filter (50 Hz) were applied, and the EMG signal was rectified and smoothed with an analog resistor-capacitor filter (time constant 10 ms) ^1^. Epochs were created for each trial from 50 ms before to 200 ms after probe onset. Each epoch was visually checked for artifacts and the peak of the startle response was marked manually. When the peak amplitude reaches two times the standard deviation above the mean baseline EMG it was considered as a valid trial. Startle trials that do not meet this criterion are treated as non-response trials. Following that, we visually evaluated the quality of baseline EMG activity.  If baseline EMG activity is stable, the quality of these trials was encoded as 1. If the baseline was relatively unstable, the quality of trial was encoded as zero. Only the high-quality trials were included to the analysis. Startle amplitude was defined as the difference between the peak startle and mean baseline amplitude (within 50 ms before the probe onset). The raw startle amplitudes were then T-standardized (mean = 50, *SD* = 10) on subject level for each session to reduce the inter- and intraindividual differences and increase the comparability across participants and across sessions (Blumenthal et al., [2005](https://onlinelibrary.wiley.com/doi/10.1111/psyp.13286%23psyp13286-bib-0004)). Trials with a startle amplitude exceeding ±2 SDs from session specific subject means were excluded from the analysis (group mean of the number of trials defined as outlier per session: baseline: 1.19 ± 0.82; post-placebo: 1.33 ± 0.76; post-ketamine: 1.38 ± 0.83). In total, 26.85 % of all trials were excluded from the analysis due to no startle response, excessive noise or additional eyeblink reflexes occurring just before or after the acoustic probe and extreme values. After the exclusion of trials, 3 sessions belonging to different participants had to be excluded because of the lack of a valid trial per picture category. Also, the placebo sessions of two participants had to be excluded due to corrupted EMG data.

**Structural and functional magnetic resonance imaging data preprocessing**

Anatomical and functional MRI data was preprocessed by means of fMRIPrep 20.1.1 ^2^; RRID:SCR_016216), which is based on Nipype 1.5.0; ^3^; RRID:SCR_002502).

Preprocessing of the anatomical MRI data was performed with the following steps: for every participant l 4 T1-weighted (T1w) images were acquired. From those images, an individualized average T1w image was calculated by means of CAT12 (CAT12 in SPM12, http://www.neuro.uni-jena.de/cat/). This average T1w image was then corrected for intensity non-uniformity and subsequently used as the T1w reference for the4 rs-fMRI sessions. Nonlinear registration with antsRegistration (ANTs 2.2.0) was used for volume-based spatial normalization to the standard space (MNI152NLin2009cAsym).

For all rsfMRI data (four sessions per participant) the identical preprocessing pipeline was performed. First, a B0-nonuniformity map was estimated based on a phase-difference map calculated by means of a dual-echo gradient-recall echo (GRE) sequence and subsequently co-registered to the generated subjective EPI references in fMRIPrep. Based on the estimated susceptibility distortion, a corrected EPI reference was calculated, which was then co-registered with the anatomical reference. Afterward, the EPIs were slice-time corrected. Head-motion parameters according to the EPI reference (transformation matrices, and six corresponding rotation and translation parameters) were calculated. Mean framewise displacement (FD) was calculated and compared between sessions. Paired t-tests revealed no significant difference in head motion between the sessions (p’s>0.05). Using the denoised EPI images, several confounding time-series were calculated based on the preprocessed images: FD, spatial standard deviation of the data after temporal differencing (DVARS) and the mean signals within the cerebrospinal fluid (CSF) and white matter (WM) (Power et al. (2014)). Confounding time-series were used for the nuisance regression for head motion and physiological signals by xcpEngine (version 1.2.2) ^4^. The volumes with excessive motion (FD> 0.25) were identified and scrubbed from the data. Prior to the nuisance regression, a temporal filter (0.01-0.08 Hz) was applied both to the confound time series and the preprocessed rsfMRI data to avoid re-introduction of filtered frequencies (Hallquist et al., 2013). Five participants who exceeded a mean FD of 0.3 mm in one of the sessions were excluded from the analysis. Single volumes were scrubbed for the motion artifacts. All participants had less than 50% censured volumes and at least ~5 min of data remained after scrubbing. In total, 30 subjects were included in the rsfMRI region of interest analysis. The PnC and CMA masks (see SFigure 2) were used to extract the mean time series and rsFC was calculated by Pearson correlation. The correlation coefficients were Fisher Z-transformed.


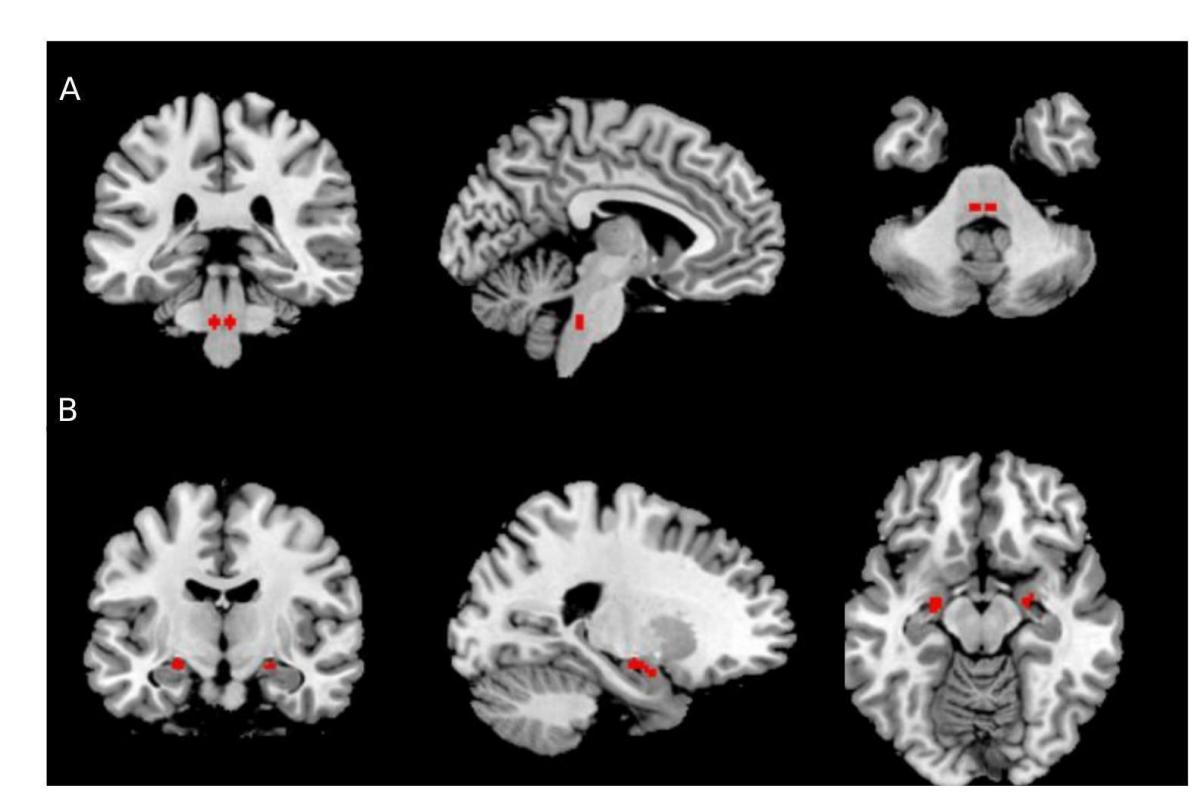


**SFigure 2**: *PnC and CMA masks A) The left and right PnC masks drawn around the peak coordinate (4 -34 -37) from Kuhn et al., 2020. B) The left and right CMA masks were obtained from the JuBrain Anatomy Toolbox v3.0*

**Supplementary Results**

**Self-reported unpleasant and pleasant affect scores**

**STable6**

*The linear mixed model fixed effect estimates for Positive affect.*

|  | B | CI (95%) | T | p | df |
| --- | --- | --- | --- | --- | --- |
| Intercept) | 6.81 | 0.62 – 13.00 | 2.24 | **0.032** | 33.00 |
| Session [Ketamine] | 0.16 | -0.96 – 1.28 | 0.29 | 0.777 | 33.00 |
| Baseline | 0.58 | 0.32 – 0.84 | 4.58 | **<0.001** | 34.00 |
| Observations | 70 | | | | |
| Marginal R^2^ / Conditional R^2^ | 0.251 / 0.655 | | | | |

B= Estimates; CI= confidence interval; Reference category for Session was Placebo

**STable 7**

*The linear mixed model fixed effect estimates for Negative affect.*

|  | B | CI (95%) | T | p | df |
| --- | --- | --- | --- | --- | --- |
| (Intercept) | 11.16 | 6.95 – 15.36 | 5.39 | **<0.001** | 33.00 |
| Session [Ketamine] | 0.60 | -0.33 – 1.54 | 1.31 | 0.198 | 33.00 |
| Baseline | 0.36 | 0.14 – 0.59 | 3.27 | **0.003** | 34.00 |
| Observations | 70 | | | | |
| Marginal R2 / Conditional R2 | 0.153 / 0.565 | | | | |

B= Estimates; CI= confidence interval; Reference category for Session was Placebo

**Subjective valence and arousal ratings of affective pictures**

**STable 8**

*Mean valence score from subjects for each picture category across session*

|  | Baseline  Mean ± SD | Placebo  Mean ± SD | Ketamine  Mean ± SD |
| --- | --- | --- | --- |
| Pleasant | 6.63 ± 0.72 | 6.63 ± 0.66 | 6.68 ± 0.74 |
| Neutral | 5.36 ± 0.36 | 5.47 ± 0.55 | 5.23 ± 0.34 |
| Unpleasant | 2.75 ± 0.72 | 2.86 ± 0.74 | 2.78 ± 0.63 |

SD: standard deviation

**STable 9**

*Mean arousal score from subjects for each picture category across session*

|  | Baseline  Mean ± SD | Placebo  Mean ± SD | Ketamine  Mean ± SD |
| --- | --- | --- | --- |
| Pleasant | 5.61 ± 1.24 | 5.14 ± 1.36 | 5.24 ± 1.56 |
| Neutral | 3.82 ± 1.15 | 3.49 ± 1.26 | 3.60 ± 1.23 |
| Unpleasant | 6.85 ± 1.55 | 6.93 ± 1.31 | 6.63 ± 1.77 |

SD: standard deviation

**STable 10**

*The linear mixed model fixed effect estimates of session and picture category on subjective valence ratings (SAM-A)*

|  | B | CI (95%) | T | p | df |
| --- | --- | --- | --- | --- | --- |
| (Intercept) | 2.76 | 2.51 – 3.00 | 22.16 | **<0.001** | 248.00 |
| Session [Ketamine] | 0.03 | -0.16 – 0.22 | 0.30 | 0.76 | 248.00 |
| Session [Placebo] | 0.10 | -0.09 – 0.30 | 1.07 | 0.29 | 248.00 |
| Picture Category [Neutral] | 2.60 | 2.29 – 2.92 | 16.48 | **<0.001** | 248.00 |
| Picture Category [Pleasant] | 3.87 | 3.46 – 4.29 | 18.36 | **<0.001** | 248.00 |
| Session [Ketamine] *  Picture Category [Neutral] | -0.16 | -0.43 – 0.11 | -1.16 | 0.25 | 248.00 |
| Session [Placebo] *  Picture Category [Neutral] | 0.00 | -0.27 – 0.27 | 0.02 | 0.99 | 248.00 |
| Session [Ketamine] *  Picture Category [Pleasant] | 0.02 | -0.25 – 0.29 | 0.16 | 0.87 | 248.00 |
| Session [Placebo] *  Picture Category [Pleasant] | -0.10 | -0.38 – 0.17 | -0.75 | 0.45 | 248.00 |
| Observations | 288 | | | | |
| Marginal R2/ Conditional R2 | 0.869 / 0.948 | | | | |
| B= Estimates; CI= confidence interval; Reference category for Session was Baseline | | | | | |

**STable 11**

*The linear mixed model fixed effect estimates of session and picture category on subjective arousal ratings (SAM-B)*

|  | b | CI (95%) | T | p | df |
| --- | --- | --- | --- | --- | --- |
| (Intercept) | 14.85 | 14.29 – 15.41 | 52.36 | **<0.001** | 240.00 |
| Session [Ketamine] | -0.21 | -0.59 – 0.16 | -1.12 | 0.26 | 240.00 |
| Session [Placebo] | -0.04 | -0.42 – 0.34 | -0.21 | 0.83 | 240.00 |
| Picture Category [Neutral] | -3.11 | -3.61 – -2.62 | -12.40 | **<0.001** | 240.00 |
| Picture Category [Pleasant] | -1.30 | -1.88 – -0.71 | -4.36 | **<0.001** | 240.00 |
| Session [Ketamine] *  Picture Category [Neutral] | 0.01 | -0.52 – 0.55 | 0.05 | 0.96 | 240.00 |
| Session [Placebo] *  Picture Category [Neutral] | -0.27 | -0.81 – 0.27 | -0.99 | 0.32 | 240.00 |
| Session [Ketamine] *  Picture Category [Pleasant] | -0.16 | -0.69 – 0.38 | -0.58 | 0.56 | 240.00 |
| Session [Placebo] *  Picture Category [Pleasant] | -0.43 | -0.96 – 0.11 | -1.56 | 0.12 | 240.00 |
| Observations | 280 | | | | |
| Marginal R2 / Conditional R2 | 0.454 / 0.848 | | | | |

B= Estimates; CI= confidence interval; Reference category for Session was Baseline

**The effect of ketamine administration on pleasant and unpleasant modulation of the startle reflex**

**STable 12**

*The linear mixed model fixed effect estimates of Session on unpleasant modulation.*

|  | B | CI (95%) | T | p | df |
| --- | --- | --- | --- | --- | --- |
| Intercept) | 1.40 | -0.42 – 3.23 | 1.54 | 0.13 | 58.00 |
| Session [Placebo] | -0.49 | -3.32 – 2.34 | -0.35 | 0.73 | 58.00 |
| Session [Ketamine] | -0.00 | -2.58 – 2.58 | -0.00 | 01.00 | 58.00 |
| Observations | 92 | | | | |
| Marginal R2 / Conditional R2 | 0.002 / 0.669 | | | | |

B= Estimates; CI= confidence interval, Reference category for Session was Baseline

**STable 13**

*The linear mixed model fixed effect estimates of Session on pleasant modulation.*

|  | B | CI (95%) | T | p | df |
| --- | --- | --- | --- | --- | --- |
| (Intercept) | -2.71 | -4.40 – -1.03 | -3.22 | **0.01** | 58.00 |
| Session [Placebo] | 1.02 | -1.63 – 3.68 | 0.77 | 0.44 | 58.00 |
| Session [Ketamine] | 2.86 | 0.57 – 5.14 | 2.50 | **0.01** | 58.00 |
| Observations | 92 | | | | |
| Marginal R2 / Conditional R2 | 0.061 / 0.099 | | | | |

B= Estimates; CI= confidence interval; Reference category for Session was Baseline

**The effect of a single S-ketamine administration on affective modulation of the raw startle reflex amplitudes**

In addition to the analysis with T-normalized startle amplitudes reported in the manuscript, we fit linear mixed models by using raw startle amplitudes as dependent variables. The main effects of Picture Category (pleasant, neutral and unpleasant) and Session (baseline, post-placebo and post-ketamine), as well as their interaction, were included as fixed terms in the model while Picture Category by Participant served as random terms. The model with raw startle amplitudes did not converge. To further visualize the affective modulation of startle reflex, the mean raw startle amplitudes were plotted across picture categories in each session (Figure S3).


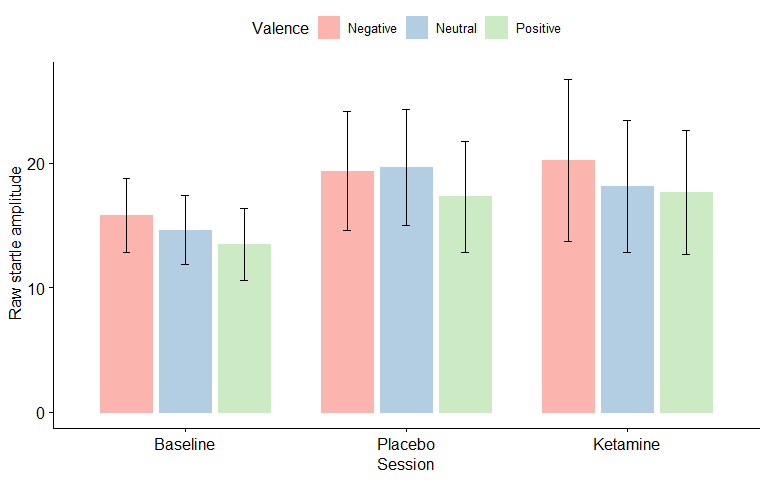


Figure S3: Mean raw startle reflex amplitudes per picture category in each session. Error bars represent standard error of the mean.

**The effect of a single S-ketamine administration on overall startle reactivity and habituation profiles**

To examine the effect of ketamine on overall startle reactivity, we calculated the mean log-transformed raw startle amplitude values per session without taking the effect of picture category into account (Fig S4). A linear mixed model was fitted, and the effect of Session (baseline, post-placebo and post-ketamine) was not significant (Table S14).

Moreover, we examined the effect of Session (baseline, post-placebo and post-ketamine) on habituation profiles by comparing the mean T-standardized amplitudes of the first three habituation trials with the mean T-standardized amplitudes of the following three as well as the last three experiment trials (Fig S5). We run an ANOVA with Block (habituation trials, first experimental block, last experimental block), Session (baseline, post-infusion placebo, post-infusion ketamine), and their interaction without taking the effect of the picture category into account. There was a significant effect of Block, while neither Session nor an interaction effect was found (Table S15).


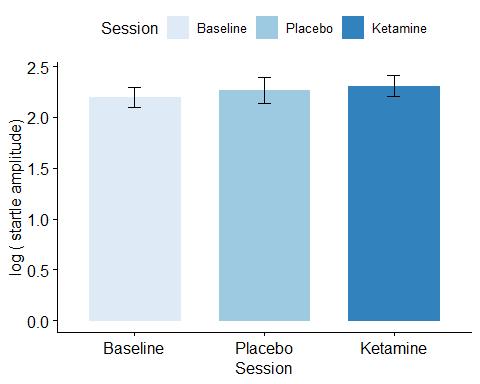


Figure S4: Mean log-transformed startle reflex amplitudes in each session. Error bars represent standard error of the mean.

**STable 14**

*The effect of a single S-ketamine administration on general reactivity of startle response*

|  |  | | | | |
| --- | --- | --- | --- | --- | --- |
|  | B | CI (95%) | T | p | df |
| (Intercept) | 2.25 | 1.92 – 2.58 | 13.39 | **<0.001** | 243.00 |
| Session [Placebo] | -0.03 | -0.23 – 0.18 | -0.26 | 0.795 | 243.00 |
| Session [Ketamine] | 0.05 | -0.15 – 0.25 | 0.44 | 0.657 | 243.00 |
| Observations | 277 | | | | |
| Marginal R2 / Conditional R2 | 0.001 / 0.605 | | | | |

B= Estimates; CI= confidence interval; Reference category for Session was Baseline


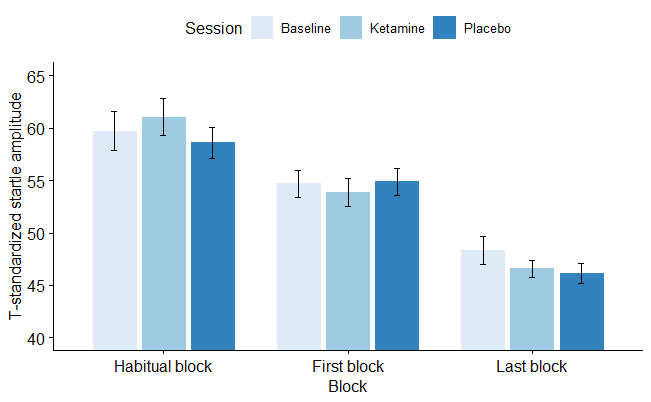


Figure S5: Mean T-standardized startle reflex amplitudes in each session. Error bars represent standard error of the mean.

**STable 15**

*The comparison of the mean startle reflex amplitudes of first three habituation trials with the mean of following three as well as last three experiment trials*

|  | *Df* | *Sum Sq* | *Mean Sq* | *F value* | *Pr(>F)* |
| --- | --- | --- | --- | --- | --- |
| Block | *2* | *6690* | *3345* | *58.894* | ***<2e-16*** ***** |
| Session | *2* | *46* | *23* | *0.403* | *0.669* |
| Block * Session | *4* | *136* | *34* | *0.598* | *0.665* |
| Residuals | *246* | *13971* | *57* |  |  |

**Effect of order and age on the ketamine's effect on affective-modulation of startle reflex and rsFC between CeA and PNC**

There was a significant effect of Picture Category (pleasant, neutral, and unpleasant) and Session-by-Picture Category interaction (Table 1) on the T-standardized startle amplitudes one day after the infusion. There was a statistically significant effect of neither treatment order (p = 0.86) nor age (p = 0.80).

There was a significant effect of Session (post-placebo and post-ketamine) on rsFC between left CMA and left PnC 24h after infusion (p = 0.04, Table 2) but not on the right side (p = 0.41). There was a statistically significant effect of neither treatment order (p = 0.69) nor age (p = 0.75).

**The frequency of valid startle responses among picture categories across sessions**

Due to the high rate of missing startle responses in our data, we examined whether the picture category or session has an effect on the frequency of the startle responses. We first calculated the frequency percentage of valid startle trials per picture category for each session (the number of valid startle trials divided by the number of presented startling probes *100, sTable16). A linear mixed model was applied using *Picture Category* (pleasant, neutral and unpleasant) and *Session* (baseline, post-placebo and post- ketamine) as fixed terms and *Participant* as a random term (see Stable). Post hoc comparisons did not show any significant difference among picture categories between or within sessions.

**STable 16**:

*The frequency of valid startle responses for each session*

| Session | Picture Category | Mean | SD |
| --- | --- | --- | --- |
| Baseline | Unpleasant | 77.96 | 22.31 |
|  | Neutral | 75.27 | 24.01 |
|  | Pleasant | 72.92 | 25.04 |
| Placebo | Unpleasant | 76.11 | 27.04 |
|  | Neutral | 77.59 | 24.51 |
|  | Pleasant | 75.55 | 24.56 |
| Ketamine | Unpleasant | 79.03 | 17.46 |
|  | Neutral | 72.39 | 24.90 |
|  | Pleasant | 75.52 | 23.37 |

SD: standard deviation

**STable 17**:

*The linear mixed model fixed effect estimates of picture category and Session on frequency of valid startle responses.*

|  | B | CI (95%) | T | P | df |
| --- | --- | --- | --- | --- | --- |
| (Intercept) | 9.39 | 8.38 – 10.40 | 18.30 | **<0.001** | 267.00 |
| Picture Category [Neutral] | -0.32 | -1.40 – 0.75 | -0.59 | 0.56 | 267.00 |
| Picture Category [Pleasant] | -0.64 | -1.71 – 0.43 | -1.17 | 0.24 | 267.00 |
| Session [Placebo] | -0.31 | -1.39 – 0.78 | -0.55 | 0.58 | 267.00 |
| Session [Ketamine] | 0.02 | -1.05 – 1.10 | 0.04 | 0.96 | 267.00 |
| Picture Category [Neutral] *  SessionID [Placebo] | 0.34 | -1.20 – 1.88 | 0.43 | 0.66 | 267.00 |
| Picture Category [Pleasant] *  Session [Placebo] | 0.57 | -0.95 – 2.10 | 0.74 | 0.46 | 267.00 |
| Picture Category [Neutral] *  Session [Ketamine] | -0.40 | -1.92 – 1.11 | -0.52 | 0.60 | 267.00 |
| Picture Category [Pleasant] *  Session [Ketamine] | 0.29 | -1.22 – 1.80 | 0.37 | 0.71 | 267.00 |
| Observations | 278 | | | | |
| Marginal R2 / Conditional R2 | 0.006 / 0.440 | | | | |

B= Estimates; CI= confidence interval; Reference category for Session was Baseline

| Session | SAM-A (%) | SAM-B (%) |
| --- | --- | --- |
| Baseline | 1.86 | 9.30 |
| Ketamine | 1.39 | 6.42 |
| Placebo | 0.95 | 6.16 |

**STable 18**:

*The percentage of the missing SAM-A and SAM-B response.*

SAM= Self-Assessment Manikin

**References**

1 Blumenthal TD, Cuthbert BN, Filion DL, Hackley S, Lipp OV, Boxtel AV. Committee report: Guidelines for human startle eyeblink electromyographic studies. *Psychophysiology* 2005; **42**: 1–15.

2 Esteban O, Markiewicz CJ, Blair RW, Moodie CA, Isik AI, Erramuzpe A *et al.* fMRIPrep: a robust preprocessing pipeline for functional MRI. *Nature Methods* 2019; **16**: 111.

3 Gorgolewski K, Burns CD, Madison C, Clark D, Halchenko YO, Waskom ML *et al.* Nipype: A Flexible, Lightweight and Extensible Neuroimaging Data Processing Framework in Python. *Front Neuroinform* 2011; **5**. doi:10.3389/fninf.2011.00013.

4 Ciric R, Wolf DH, Power JD, Roalf DR, Baum GL, Ruparel K *et al.* Benchmarking of participant-level confound regression strategies for the control of motion artifact in studies of functional connectivity. *NeuroImage* 2017; **154**: 174–187.
